# Desert Sand and Dust Storms and Desert Dust Episodes: Major Patterns to be Accounted for to Protect the Health of Exposed Population: A Review

**DOI:** 10.1021/acsestair.5c00201

**Published:** 2025-11-24

**Authors:** Xavier Querol, Julia C. Fussell, Najat A. Saliba, Ali Al-Hemoud, Kari C. Nadeau, Aurelio Tobías, Masahiro Hashizume, Mazen Malkawi, Sophie P. Gumy, Kerolyn K. Shairsingh, Pierpaolo Mudu, Philip K. Hopke

**Affiliations:** 1 Institute of Environmental Assessment and Water Research (IDAEA), Spanish National Research Council (CSIC), Barcelona 08034, Spain; 2 Environmental Research Group, Medical Research Council (MRC) Centre for Environment and Health, School of Public Health, 4615 Imperial College London, London W12 0BZ, United Kingdom; 3 Member of the Lebanese Parliament, Beirut VGW3+QJ3, Lebanon; 4 Kuwait Institute for Scientific Research, Environment and Life Sciences Research Centre, Safat 13109, Kuwait; 5 Department of Environmental Health, Harvard T.H Chan School of Public Health, Boston, Massachusetts 02115, United States; 6 Department of Global Health Policy, Graduate School of Medicine, 13143 The University of Tokyo, Tokyo 113-0033, Japan; 7 Environmental Health Advisor at the Ministry of Health and Prevention, Dubai 1853, UAE; 8 Department of Environment, Climate Change and Health, World Health Organization, Geneva 1211, Switzerland; 9 World Health Organization, Regional Office for Europe, European Centre for Environment and Health, Bonn D-53113, Germany; 10 Institute for a Sustainable Environment, Clarkson University, Potsdam, New York 13699, United States; 11 Departments of Public Health Sciences and Environmental Medicine, University of Rochester School of Medicine and Dentistry, Rochester, New York 14642, United States

**Keywords:** air quality, health impact, desert dust

## Abstract

Sources of desert dust, atmospheric transport, recorded concentrations of atmospheric particulate matter (PM), physical, compositional, and biological characteristics, and likely direct and indirect impacts on air quality impairment are reviewed without a systematic, but with an expert approach. The aim is to offer information necessary to protect the health of exposed populations in the dust-emitting and dust-receptor regions. This review corroborates the complexity of the process by which air quality is impaired during dust episodes, the mixture of components that PM might contain during dust episodes, the differences between dust emission and reception regions, and the interplay of indirect effects (thinning the boundary layer; concentration of local pollution; interactions with anthropogenic pollutants). Based on these dust episode patterns, we recommend the implementation of alert systems to protect the more vulnerable members of the population and highlight a number of recommendations for air quality monitoring during such episodes to provide adequate data sets to rigorously evaluate health outcomes associated with dust exposure in emitting and receptor regions and the possible causes for these effects.

## Graphical abstract

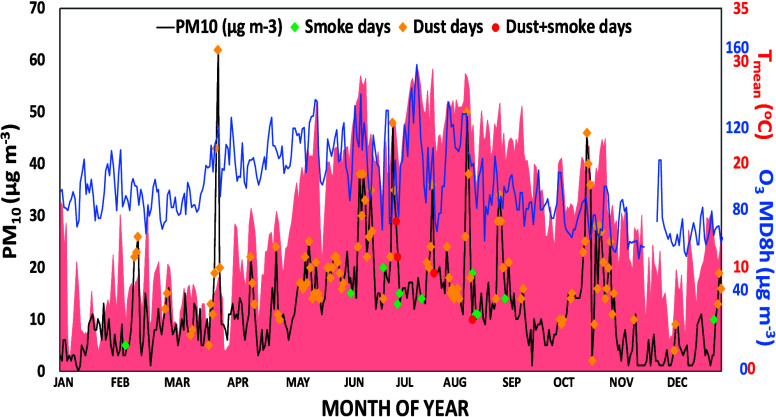

## 1 Introduction

This review focuses on desert dust patterns relevant to assessing the effects of exposure to high concentrations of this atmospheric pollutant on human health. It does not have a meteorological and mechanistic focus. Thus, the definitions given below are adapted to the air quality focus of this article.

**‘Desert dust’** is particulate matter (PM) that is emitted from the surface of arid and semiarid regions by wind moving over it. Desert dust particles must have relatively fine diameters to be suspended and transported across large distances (e.g., clay and silt size fractions, < 2 and 50 μm, respectively). The term ‘**Dust’** includes a variety of PM sources that have a dominant (but not exclusive) mineral composition, such as desert dust, road dust, construction dust, industrial and ‘**Agricultural dust**’, the latter being dust anthropogenically emitted by agricultural activities. ‘**Soil dust**’ is also used in the literature (but not in this review), as a general term for natural and anthropogenic emissions of dust. **‘Sand’** scientifically refers to the sediment fraction with a size of 50–2000 μm. Sand has a very relevant role in generating dust by eroding surfaces and emitting dust particles, and in the saltation of sand particles and breakdown into dust particles.^2^ The term **‘sand and dust storms’ (SDS)** usually refers to the meteorological phenomenon that occurs when strong winds lift large amounts of sand and dust from bare, dry surfaces into the atmosphere.^1^ **‘Storm’ (in SDS)** refers to the mechanism by which high winds dislodge particles and carry them into the air.^1^ However, **SDS in this review**, refers to high, local concentrations of PM in emitting areas, but not for downwind receptor ones, which are only affected by fine transported dust.

The arid nature of desert dust emitting regions results in soils with a low organic content and therefore, the emitted PM is primarily mineral in content. According to a number of classic studies,^3–6^ large emitting regions are often inland basins occasionally flooded by ephemeral surface water. High winds can suspend minerals deposited by washout-deposition processes, causing SDS. Frequent nocturnal thermal inversions, in the emission area (defined below), keep a ‘**dust laye**r’ above the boundary layer where winds of the midtroposphere are intense, transporting desert dust for thousands of kilometres (‘long-range transport’) from the source causing ‘**desert dust episodes**’ **(DDE)** in receptor regions (defined below). In this review, we refer to ‘**episode or event**’ as a short-term event characterized by unusually high concentrations of dust.

The processes of emission and transport are not generally continuous although large regions such as North Africa and The Middle East experience desert dust activity quasi-permanently throughout the year. The episodes are caused by desert dust emission from a source area, or ensemble of source areas, followed by transport to a receptor region. In this air quality framework, an ‘**emission area’** is an area very close to the emission of desert dust, where PM can reach extremely high concentrations. A **‘receptor region’** is a region located close or far away from the emission source where air quality is affected by desert dust transport. In this review, the term ‘**DDE**’ describes a desert dust event in the receptor region only, while SDS, as noted above, refers to that in the emission area.

During SDS and DDE, a mixture of PM from desert dust and anthropogenic sources occurs in varying proportions. In receptor regions, desert dust is typically mixed with locally and/or regionally emitted PM.^7^ This type of mixing might also occur during transport toward the receptor region when high-desert dust air masses cross polluted areas, and in the source area when desert dust is emitted from polluted sediments and soils or mixed with local air pollutants.^8–10^ Deposited desert dust with a high load of anthropogenic pollution is continuously resuspended. Broomandi et al.^8^ found soils in Southern Iraq that were highly enriched with specific pollutants from different anthropogenic inputs, including wars.

There are many reviews and books on the nature and environmental impacts of desert dust including phenomenology of episodes, particle size and composition, as well as health effects of dust storms in different regions.^2–4,11–31^ Experimental research also describes mechanistic pathways underlying the pathogenesis of human respiratory disorders, thereby supporting epidemiological findings by demonstrating biological plausibility for events including exacerbation of asthma, hospitalization for respiratory infections and seasonal allergic rhinitis.^32^ However, there remains a need to understand the health impacts of desert dust exposures in both emission and receptor regions owing to distinct differences in these two domains (e.g., with respect to intensity, anthropogenic content of PM, meteorological patterns, etc.). A report produced by the World Health Organization (WHO^33^) in 2019, supporting the update of the 2021 WHO Air Quality Guidelines,^34^ systematically reviewed the scientific evidence on SDS health effects, concluding the urgent need to harmonize the characterization of exposure to desert dust and copollutants if consistent conclusions are to be drawn on the health effects associated with desert dust episodes. A published overview^26^ of (a) desert dust exposure parameters for health studies, (b) criteria to implement SDS alert systems to protect public health, and (c) epidemiological analyses, found that short-term health effects reported during desert dust episodes in different regions of the world often-varied results because of differing effects of bulk PM, desert dust-PM, and nondesert dust-PM. Variations are likely caused by differences in emissions (natural and anthropogenic) patterns of desert dust, atmospheric processing, modification of composition of dust due to human activities (e.g., industrial emissions) and measurement strategies employed by epidemiological studies.^28,29^ Other more recent studies^34,35^ also reporting on desert dust health effects suggested that more PM_2.5_ and PM_10_ (particles with a size lower than 2.5 and 10 μm, respectively) exposures and health data were required to better evaluate SDS-DDE health effects.

Here we update the above-mentioned 2019 WHO report on desert dust exposure and the derived publication.^26^ The need for such a revision stemmed from (a) a WHO working group on health effects of desert dust that identified a need to implement approaches in desert dust emission and receptor regions, in terms of forecasting episodes, monitoring desert dust exposure and identifying harmful desert dust components for the protection and evaluation of health effects; (b) some changes and improvements in modeling tools used for detection and forecasting; and (c) the availability of new approaches to quantify desert dust and nondesert dust contributions. Furthermore, this review provides an opportunity to examine the relevant scientific literature published since 2019. Accordingly, the focus of this review is to provide scientific guidance on (a) SDS and DDE phenomenology and the differences in PM concentrations, sizes and composition; (b) possible compositional changes in PM associated with DDE due to anthropogenic interferences (c) updated tools for SDS and DDE monitoring and alert systems, and limitations in SDS regions; and (d) major PM and meteorological patterns that should be accounted for when evaluating health effects of desert dust exposure.

## 2 Source Regions and Emissions

Oceans and arid regions provide most of the Earth’s atmospheric aerosol load, with 6.3–10.1 and 1200–1800 Tg (10^12^g)/year­(yr) of sea salt and PM_10_ soil dust emitted into the troposphere, respectively.^5,36–39^ More recently, global dust emissions have been estimated to reach 4700 (3300–9000), 1800 (1200–2900) and 220 (190–270) Tg/year of PM_20_, PM_10_, and PM_2.5_, respectively.^40,41^ Thus, 12% of the PM_10_ emissions fall in PM_2.5_ size range However, ambient measurements report that PM_2.5_ accounts for 30% of PM_10_ dust,^42^ probably because of the longer atmospheric lifetime of the latter. Dust can be emitted from any uncovered dry soil surface under windy conditions, including agricultural land.^5^

Desert dust source regions are active throughout the year with emission frequency and intensity peaking in specific seasons. Desert dust sources have been identified empirically from satellite radiance measurements.^4,43–46^ Ginoux et al.^5^ and Klose et al.^6^ derived the global distribution of dust sources at 10 km resolution by counting the frequency of days marked by high dust optical depth (DOD). A wide band of dust emitting regions is observed from 0° to 50°N (Figure 1), with a major hotspot in Northern Africa, and some additional and less intensive ones outside of this band, such as those in South America, South Africa, and Australia).

**Figure 1:**
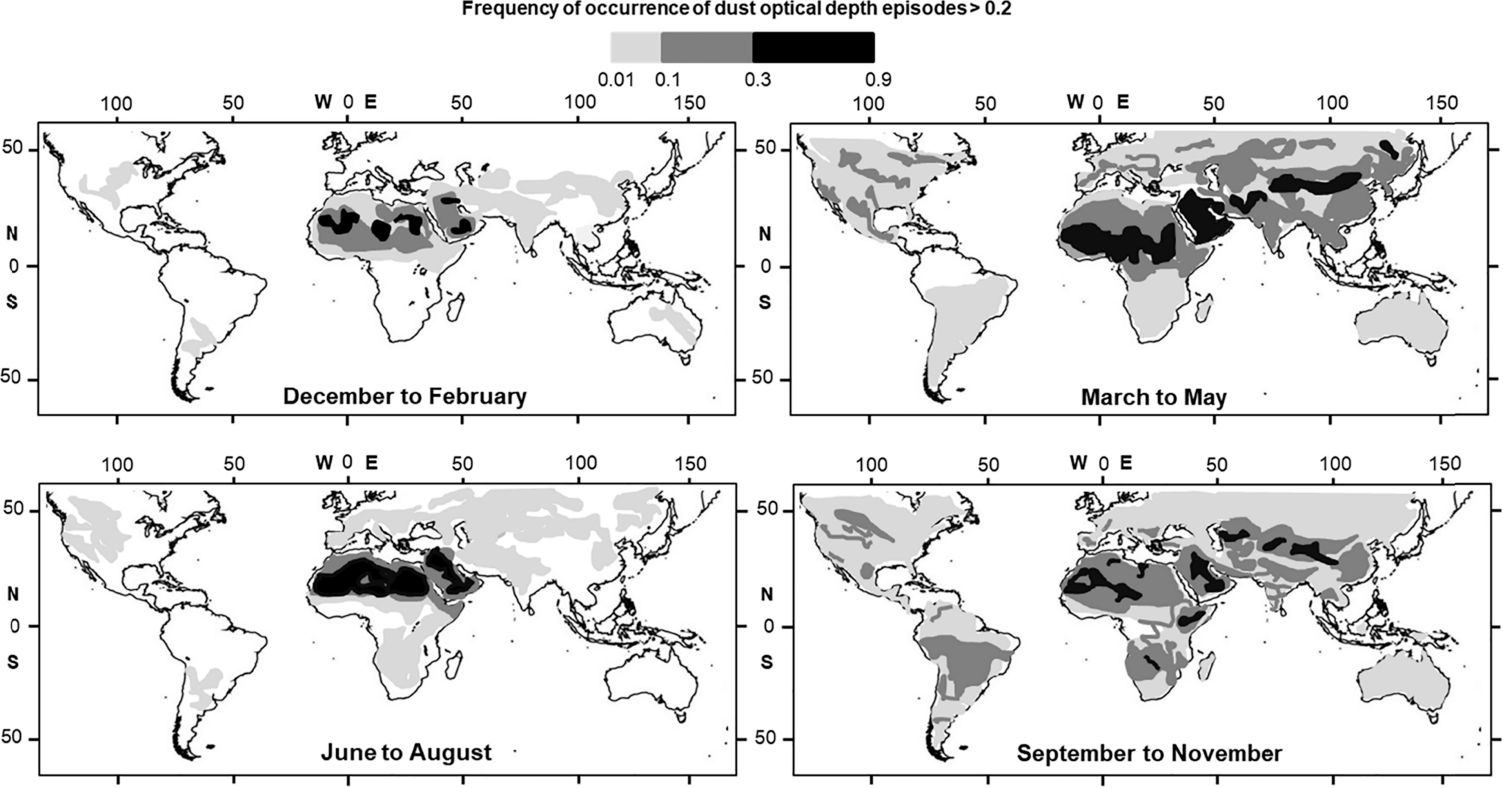
Seasonally averaged frequency of occurrence for 2012 (FoO calculated with respect to the number of days in the season) of MODIS Deep Blue Dust Optical Depth (DOD) > 0.2 (550 nm), normalized by the number of days per season. From Klose et al.^6^

The Sahara desert and Sahel region in Northern Africa are the most active sources of desert dust emissions (most probably, 790–840 Tg/yr), followed by The Middle East (120–2225 Tg/yr), Gobi and Taklamakan Deserts in eastern Asia (140–220 Tg/yr), Central Asia, Eastern Australia, South America (Atacama) and South Africa (10–60 Tg/yr each), and Southern US-Northern Mexico (2–60 Tg/yr).^4,5,38,44–46^ Although Iceland had not been considered a major natural dust source, Arnalds et al.^47^ estimated emissions of 30–40 Tg/yr, a relevant emission rate with very different mineral patterns compared to other sources. Other studies^48–51^ reported that dust emissions from Alaska, Canada, Greenland, and Iceland contribute to a relevant 100 Tg/yr fraction of global dust emissions.^49^ Examples of other sparse sources occur in specific areas of Spain and Turkey.^5,11^ Kok et al.^41^ estimated that North Africa contributes 50% of global dust and Asian sources 40% (30% Middle East and central Asia, 10% East Asia). Minor source regions contribute 10%, with 2.5% from North America, 7.5% from the Southern Hemisphere (Australia, South America, and Southern Africa). Groot Zwaaftink et al.^48^ reported around 3% for high-latitude dust emissions.

Prolific desert dust sources generally correspond to topographic depressions where a deep layer of alluvium has accumulated.^4^ Many of the most important ones are not always large regions with uniform emissions across them.^52,53^ Dry lakes with unconsolidated fine-grained sediments and ephemeral streams-rivers, lakes, and playa-lakes or sabkhas (ephemeral lakes in inland basins without connection with coastal areas), where sediments are deposited intermittently can also be prolific sources.^3^

The anthropogenic contribution to dust emission in arid regions originates from land dissection, disturbance and desiccation of lakes and playa-lakes, agricultural practices, and expansion of livestock grazing^54–59^ and ranges, owing to large modeling discrepancies, from 10%^60^ to 50%.^61^ In Australia, 76% of dust sources were associated with land use, the Middle East and Indian subcontinent 30% and East Asia during the Asian summer monsoon season 40%.^5^ At the other extreme, the fraction due to human activity is as little as 8% in Northern Hemisphere Africa, in part because of expansive natural sources within harsh desert environments that are inhospitable to cultivation.^5^

Recently, remote sensing observations reported that approximately 5% of the Earth’s continental surface emits dust, predominantly from North Africa (67%) and Asia (30%).^62^ The study revealed that global dust sources consist of desert, vegetative, and hydrological categories, representing 65%, 26%, and 9%, respectively. Among these categories, sandy terrains, rangelands, and temporary water bodies present the most extensive coverage worldwide, in that order. Contributing factors to the development of global dust sources were found to be natural (65%) and anthropogenic (35%), and a global upward trend in the frequency of dust events originating from desert areas was also found. Both natural and anthropogenic dust emissions are highly influenced by the hydrological cycle and consequently, are highly affected by climate variability.^24,25^ For example, a clear relationship between the prior year rainfall in the Sahel region and DDE occurrences in the Caribbean region was demonstrated.^63^ Severe drought in combination with poor land use have been demonstrated to contribute to the formation of SDS.^58^ With increased atmospheric and ocean temperatures from climate change and land use changes SDS frequency and intensity are expected to increase. Indeed, the analysis of 1948–2021 trends reveals a clear increasing trend for DDE over Spain.^64^ In Central Europe, Varga^65^ reported for 1979–2018, an increase in desert dust storms in the 1980s, and a decrease during the 1990s. Subsequently, the frequency increased again. For Northern Europe, Varga et al.^66^ found for 1980–2022 a similar trend for Finland, with a decline from an active period in the 1980s to the 1990s, as well as more frequent and intense episodes in recent years including a doubling of winter episodes from 2010. On a global scale, Xi^67^ reported a declining dust trend over 1984–2012 and that the mean dust episode frequency increased at 0.02%/yr from 1986 to 2019. North Africa experienced increased dust activity since 2010, due to reduced soil moisture and enhanced wind speed following the transition of the North Atlantic Oscillation from strong negative to recurring positive phases in 2011. It was also found that The Middle East had a significant decline of dust production since 2015, and East Asian dust followed a decreasing trend from 1986 to 1997, and stable frequency of episodes since then. Tang et al.^68^ reported that global dusty weather follows an interdecadal variability, with a cycle of 10–14 years.

## 3 Atmospheric Transport

Once emitted, desert dust can be transported and distributed across the planet, with wide variations in space and time. Temporal variability is evident at multiple time scales from diurnal cycles, the episode scale and the seasonal variations to interannual variability and trends that are mostly driven by meteorological factors, controlling both emission and transport processes. Figure 2 and Table 1 summarize the most relevant long-range transport and seasonal paths based on prior studies.^5,6,13,26^

**Figure 2:**
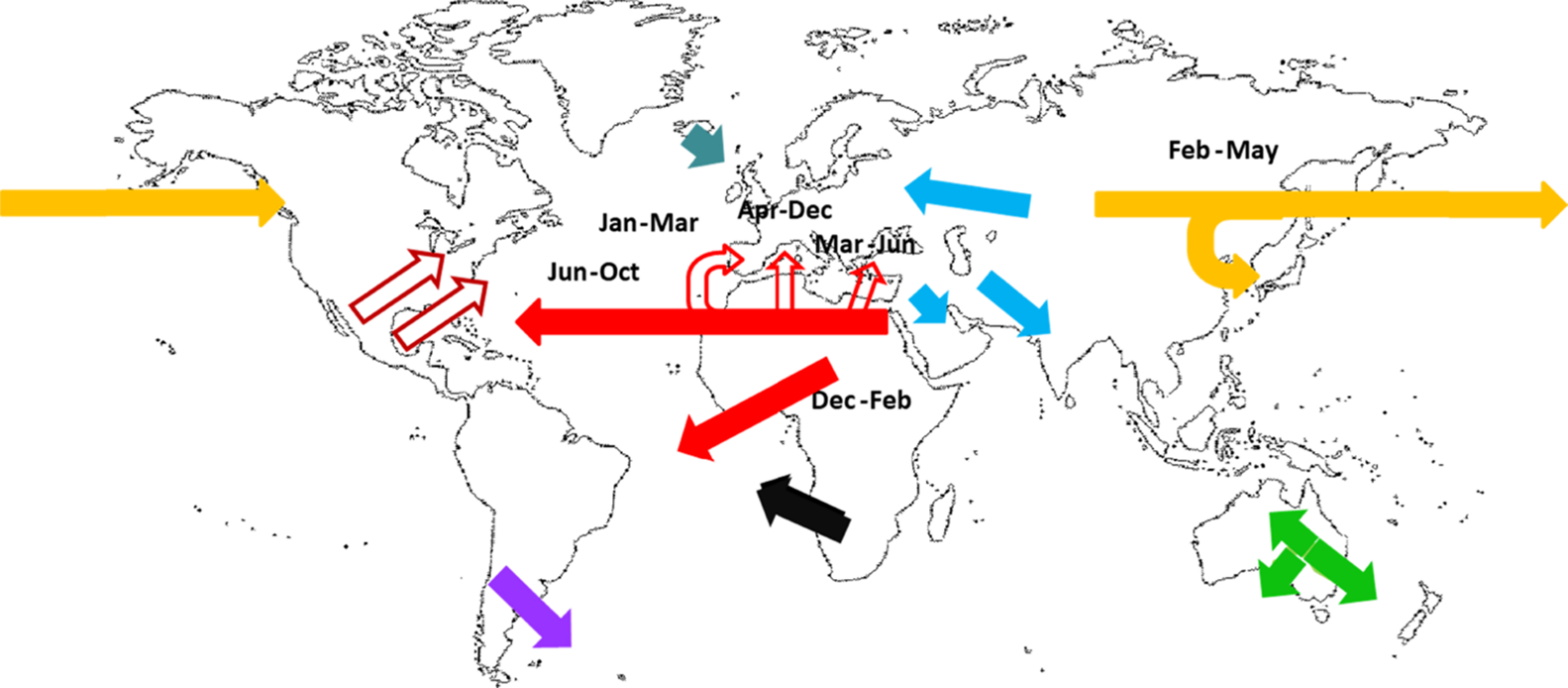
Major desert dust transport fluxes, modified from prior studies.^5,6,13,26^

**Table 1:** Major Desert Dust Pathways and Seasonal Emission Peaks

major dust pathways	
from	toward
Sahara and Sahel	Caribbean, South America and USA
Sahara	Southern Europe and Eastern Mediterranean
Iceland	Northwest Europe
Ukraine	Central Europe
Middle East	Central and Southern Asia
Taklimakan and Gobi	Pacific, reaching as far as the US
Southern USA	Mexico and Eastern USA
Mexico	Southern USA
South Africa	Atlantic
South America	Southern Atlantic
Central Australia	different surrounding regions

seasonal emission peaks	
season	region
spring and summer	Northern Africa
summer	North and South America and Southern Africa
summer and autumn	Middle East, Central Asia and Australia, and the US
autumn-winter	South America
winter	India
autumn-spring	Eastern Asia

Desert dust storms usually last between 1 to 24 h in the source area.^17^ However, high atmospheric concentrations of desert dust can persist. In desert areas convective processes inject desert dust to high altitudes where synoptic circulations can transport it over very large distances.^4,5,69,70^ At night, thermal inversions isolate upper and lower atmospheric layers, favoring continuous transport. Figure 3 shows an example of a regional impact of an African DDE over Spain in February 2016, which caused very high levels of PM_10_ and PM_2.5_ (exceeding by up to >6- and >4-fold the respective daily WHO guidelines^34^) for a few days over large regions of the country. Note that over several periods, high PM_10_ caused by desert dust and forest fire events coincided with high O_3_ levels.

**Figure 3:**
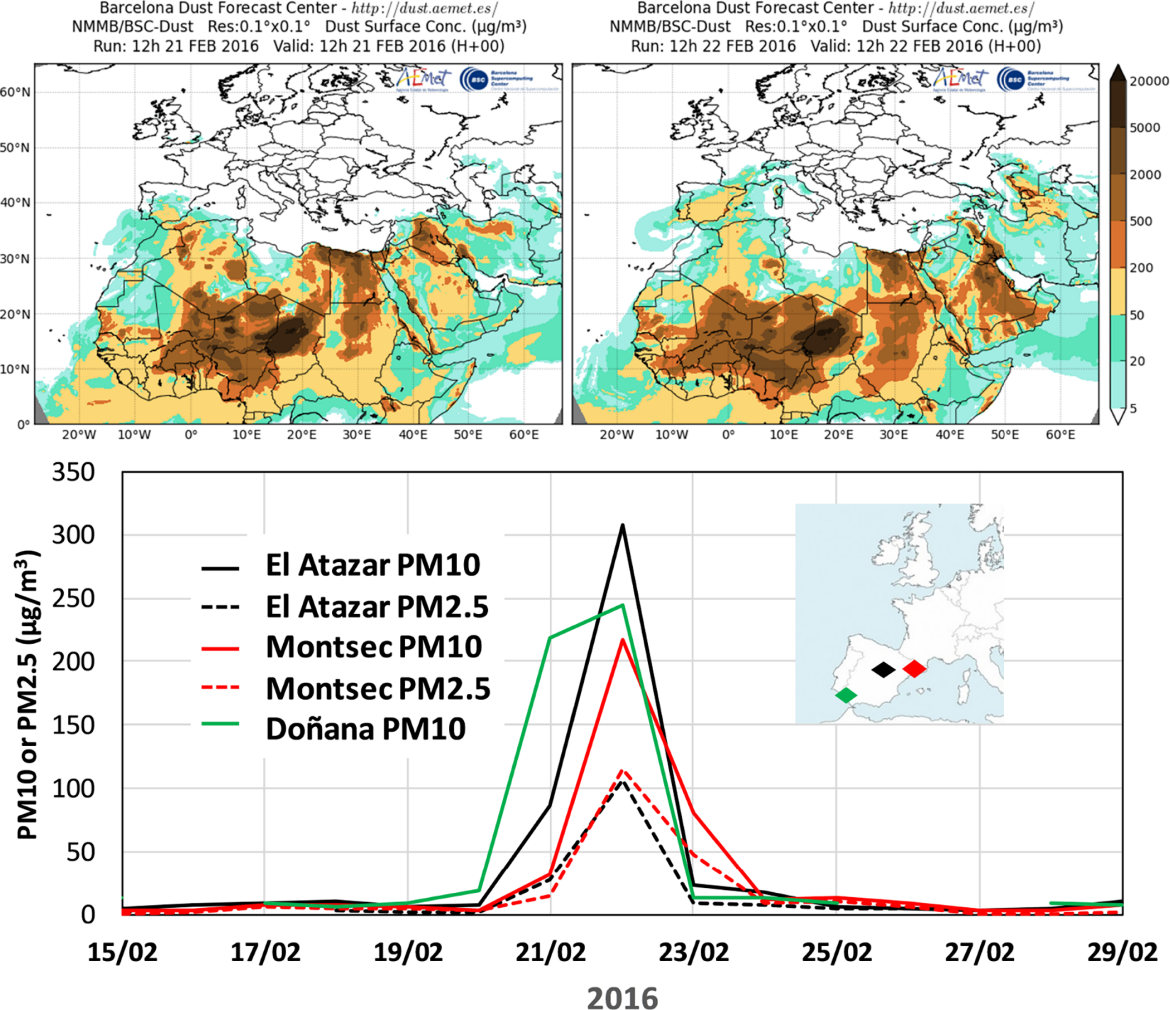
Example of a regional impact of an African DDE over Spain (top shows the surface desert dust concentrations according WMO-AEMET-BSC Dust Observation Centre, https://dust.aemet.es), reaching daily measured PM_10_ concentrations exceeding 300 μg/m^3^ in the central regions, but also exceeding 100 μg/m^3^ of PM_2.5_ (bottom) in regional background sites.

As mentioned above, depending on meteorology, desert dust can be transported near surface levels or lifted to high altitudes, becoming subject to long-range transport (Figure 4). Saharan dust is transported toward the Caribbean at surface levels in winter and at higher altitudes in summer; and in the Canary Islands in winter air quality is very often impacted by desert dust (*Calima)* and local pollution, whereas in summer, desert dust layers have less influence on surface PM concentrations.^71^ Furthermore, the planetary boundary layer (PBL) thickness can be significantly lower during desert dust storms due to desert dust layers decreasing the insolation at surface levels and convective mixing, and other desert dust-transport processes causing atmospheric subsidence or thermal inversions, resulting in increased local pollutant concentrations at the surface in receptor regions.^7,72^ These effects do not affect all emission areas.^73^

**Figure 4:**
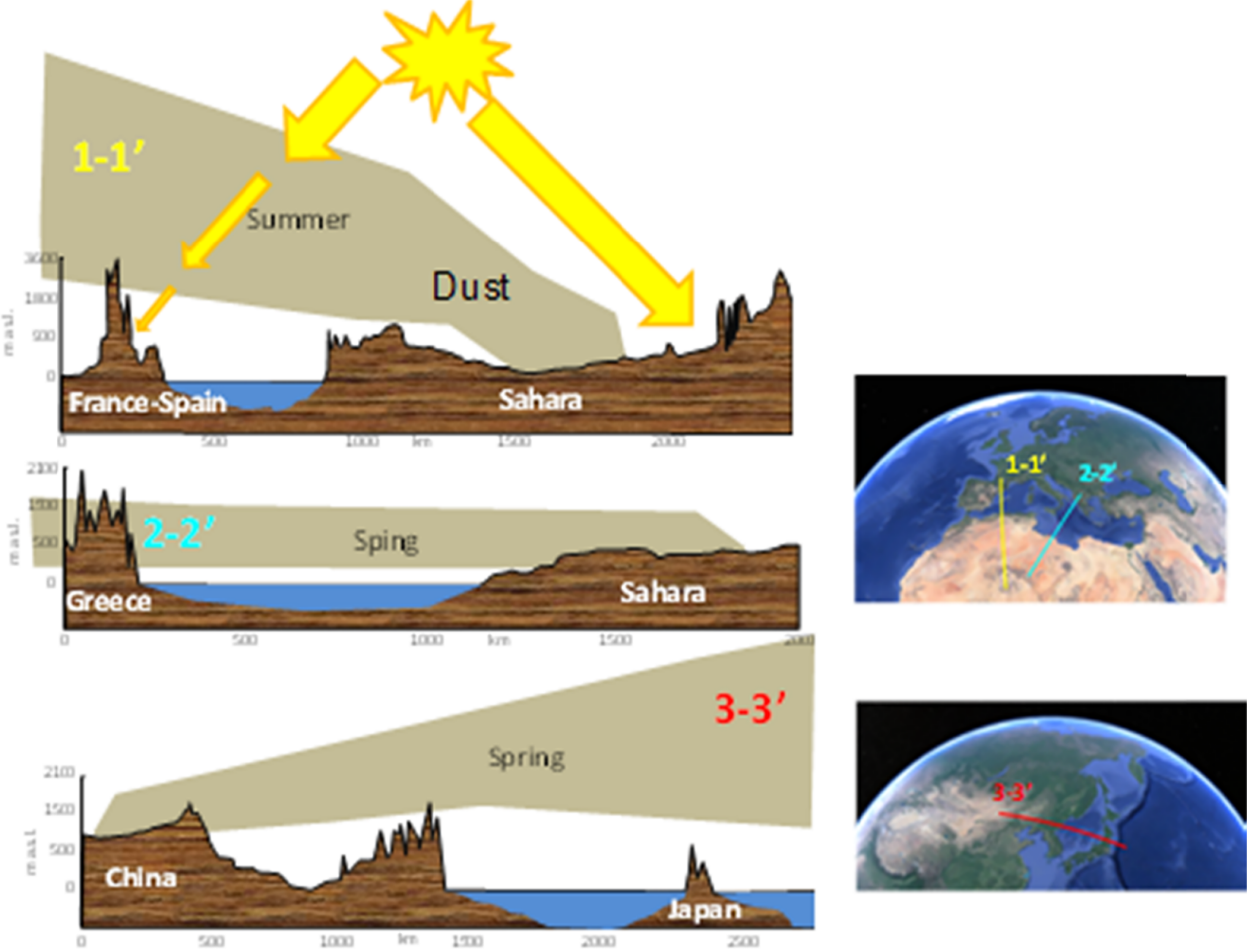
Simplified scheme showing different scenarios concerning altitude of transported desert dust layers and the impact on reducing insolation on surface. See location of 1–1′; 2–2′; 3–3′ cross sections in the right maps.

The altitude of desert dust-enriched air masses leaving Western Africa toward the Caribbean region typically ranged from 0.1 to 4–5 km.^74,75^ Over Europe, multiple desert dust layers of variable thickness (0.3–7.5 km, mean 1.5–3.4 km, depending on the European region) are transported at mean altitudes of 2.5 (base) and 5.9 km (top) and have a maximum height of 10 km.^76,77^ A 1 to 4 km desert dust layer thickness was reported over Eastern Asia and the Pacific during a yellow desert dust or *Kosa* event.^78^

Over continental areas, PBLs can reach high altitudes, and by convective mixing desert dust layers at high altitudes, might affect surface levels. This effect is very clear when observing surface desert dust maps that show low concentrations below desert dust layers over oceans, where the marine boundary layer is thin, and much higher concentrations over land. Figure S1 presents a DDE over Asia on 19 June 2021 when desert dust plumes reached the Indian Ocean.

## 4 PM Size and Concentrations

The impacts of desert dust on air quality were reported initially by studies in the 1970s to 1990,^79–83^ which described the influence of African DDE on ambient total suspended particles (TSP) concentrations in receptor regions remote from emissions. Prospero and Lamb^63^ and Prospero et al.^84^ in the early 2000s, reported a marked impact of desert dust transported from Africa on ambient PM_10_ concentrations in the Caribbean Region and Southeastern US. Multiple studies in the 2000s^85–88^ reported similar impacts of Asian desert dust on ambient PM concentrations over western US. In the late 1980 and 1990s, a number of studies reported Northern African dust impacts on ambient TSP over Europe and Turkey.^89–92^ For regional background sites in Northeastern Spain it was found in the late 1990s that most exceedances of the PM_10_ daily limit value were caused by Northern African dust events in regional background sites.^93^ Many studies have described similar findings in Spain (including the Canary Islands) and Southern Europe since the 2000s,^94–99^ Northern Africa,^100,101^ the Mexican desert region,^102,103^ Central Asia,^104^ Australia,^105^ East Asia,^106^ and the Middle East.^107,108^

While the influence of desert dust emissions is particularly pronounced for ambient TSP and PM_10_, PM_2.5_ concentrations also peak, especially during strong events, given the very high concentrations that can be reached during SDS, especially in proximity of source areas, but also at distant regions.^109,110^

### 4.1 PM Size

Mahowald et al.^111^ and Reid et al.^112^ reported that volume particle size desert dust distribution for particles <5 μm is quite similar in different desert regions of the world, while major differences occur in the >5 μm fraction, due to variations in the state and particle size distribution of emitting soils and sediments. At emission areas, volume particle size distributions usually have a bimodal shape, with modes at 7–5 μm (minor) and 3–4 μm (dominant). The impact of pollution is also evident at times, with the upswing in particle volume for <0.8 μm aerodynamic diameter.^113^ As the transport distance increases, the coarser mode markedly decreases as a consequence of differential deposition. Airborne measurements of volume particle size distributions of the African dust layer transported over the Western Mediterranean reported a median aerodynamic diameter of 3–4 μm, with three particle size volume modes distributions of 0.2, 4 (dominant) and 30 μm in modal diameter.^113^ The occurrence of the very coarse desert dust particles transported over 1000 km is not well understood according to these authors.

Median mass diameter of desert dust reaches 3.5 ± 1.3 μm, and the aerodynamic diameter 4.3 ± 1.1 μm,^11,112^ with values ranging from 3.0 to 3.5 ± 0.5 μm (median aerodynamic diameter) in remote oceans, Barbados and Puerto Rico to 3.6–6.5 ± 1 μm (median aerodynamic diameter) in Turkey-Libya, Sahara, Arabia, Iraq, Yemen, Negev, Canary Islands, desert dust hotspots in US, Gobi-Taklamakan and Tadzhikistan. Similar modes are reported for a number of sites around the globe,^112^ and during an intense *Kosa* in Japan (with a prevalent size mass of 3.3–4.7 μm) and Beijing (4.7–7.0 μm).^106^ During a DDE affecting Australia, more than 50% of the TSP fell in the PM_10_ size fraction,^105^ and 13% of the PM_10_ fell in the PM_2.5_ fraction.^114^ A median aerodynamic diameter of 2.2 μm for DDEs affecting the Caribbean region was reported,^115^ and a higher PM_2.5_/PM_10_ ratio as the proportion of marine aerosol (3.8 μm aerodynamic diameter) increases.

Based on these above data, it seems that far from sources, a 2.0–3.5 μm mean/median mass-size is typically measured, whereas close to sources, 3.5–7.0 μm are frequently obtained (Figure 5). These values refer to the mean/median values but when high concentrations of desert dust are recorded, the finer and coarser size tails influence high PM_2.5_ and PM_10–20_ absolute concentrations.

**Figure 5:**
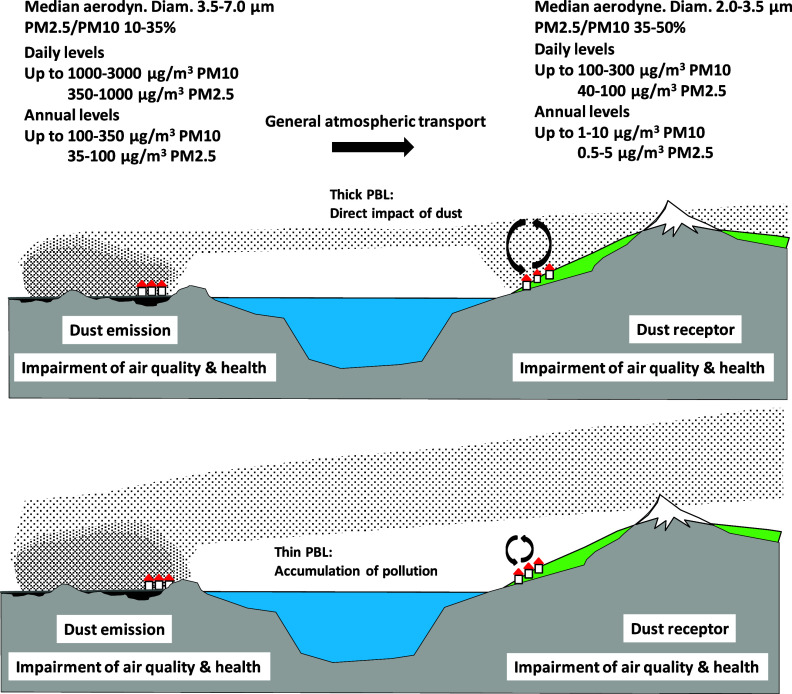
Simplified scheme showing typical desert dust size patterns and maximum daily and annual PM_10_ and PM_2.5_ concentrations reached during SDS at the emission source and receptor regions. The upper panel represents a day with a thick PBL that transports vertical desert dust to the surface. The lower panel is a day with thin PBL, which might be caused by a thick desert dust layer (reducing solar radiation affecting the surface), which in turn accumulates local pollution without the impact of desert dust.

Kandler et al.^116^ found that for proximal points and the most acute episodes in the Sahara Desert, only 3% of TSP was made of PM_10_, and <35% of PM_10_ was PM_2.5_. For other dusty days, PM_10_ to TSP proportions varied from 1 to 9%, and PM_2.5_ to PM_10_ from 15 to 36%. Goudie^17^ and Engelbrecht et al.^108^ reported data on PM_2.5_ to PM_10_ proportions from sites influenced by desert dust. Querol et al.^26^ used these data to conclude that for PM_2.5_ concentrations exceeding 100 μg/m^3^, in 92% of cases the proportion of PM_2.5_/ PM_10_ ranged between 10 and 40% (and in 67% of cases between 10 and 30%), whereas for lower PM_2.5_ concentrations, this percentage varied widely (10–60%), probably due to variability of PM sources for this lower PM_2.5_ concentration range. In another study, the same group reported PM_2.5_/PM_10_ ratios of 30% as an average for African dust days over the Canary Islands, and around 50% over mainland Spain.^110^ Based on this information, it would appear that close to the source, 10–35% of PM_10_ is PM_2.5_ in DDEs, while in receptor regions these proportions are in the range of 35–50% (Figure 5).

Data for ultrafine particles (<0.1 μm in size) during DDEs are very scarce. Jayaratne et al.,^114^ Wehner et al.,^117^ and Querol et al.^110^ reported data for desert dust episodes in Brisbane, Beijing, and Barcelona, respectively. The first two studies found decreased ultrafine particle concentrations, probably due to dispersion of local pollution and agglomeration and coagulation of locally emitted ultrafine particles on the abundant desert dust particles. The third study did not find concentrations of UFP to be affected during DDEs. However, Casquero-Vera et al.^118^ reported that at a high-altitude mountain site in Southern Spain, new particle formation events (producing high UFP concentrations) were favored during Saharan dust episodes. This phenomenon requires further investigation but might result from catalytic effects of TiO_2_ and Fe_2_O_3_ in desert dust favoring oxidation of VOCs to compounds that nucleate and form new particles.

### 4.2 PM Concentrations

A summary of studies of TSP, PM_10_, and/or PM_2.5_ are summarized in Table S1. Concentrations of up to tens of thousands μg/m^3^ of PM_10_ concentrations in extreme DDEs^106^ to several thousand in intense ones^116^ are reached in desert dust hotspots. In close-to-hotspots areas, such as the border of the Taklamakan desert, Middle East cities, Canary Islands and Cabo Verde, daily averages of a few thousands μg/m^3^, but more frequently several hundreds of PM_10_ might be reached.^107,119–122^

In receptor regions at substantial distances from emission areas, concentrations are considerably less, but still very relevant for air quality impairment. Daily PM_10_ concentrations during African dust episodes reaching several hundreds of μg/m^3^ in remote sites of Spain and Cyprus were reported.^95,110,123^ African dust annual mean contribution of 1 to 8 μg/m^3^ of PM_10_ in regions surrounding the Mediterranean were reported.^99,124^ Daily concentrations exceeding 100 μg/m^3^ of PM_10_ were reported in the Caribbean region and Barbados,^125–127^ and in Japan concentrations close to 100 μg/m^3^ PM_10_ were measured during DDEs in China.^128^

Goudie^17^ reviewed desert dust concentrations across the world, reporting maximum values of 43–86, 63–700 and 42–911 μg/m^3^ of PM_2.5_ during DDEs across Southern Europe, Eastern Asia, and other regions (Iran, Australia, US, Israel), respectively with 43–47, 11–61, and 13–40%, respectively, of the PM_10_ mass being PM_2.5_. Mean annual values from the Middle East of 72–303 and 35–111 μg/m^3^ for PM_10_ and PM_2.5_ were reported, respectively,^108^ with 21–60% of PM_10_ mass being PM_2.5_. Goudie^17^ also compiled maximal PM_10_ concentrations at receptor regions, ranging from 150 to 2500 μg/m^3^ in Southern Europe and 134 to 3006 μg/m^3^ in Japan-China-Taiwan-Korea; and at sites closer to sources, from 266 to 15366 μg/m^3^ in Australia, 312 to 5000 μg/m^3^ in Western Africa, 123 to 65112 μg/m^3^ in North America, and 700 to 5619 μg/m^3^ in the Middle East.

Figure 5 summarizes results on reviewed concentrations for PM_10_ and PM_2.5_ in emission and receptor regions. As discussed above, in and around emission source regions, daily PM_10_ concentrations may reach several thousand μg/m^3^, with PM_2.5_ contributing around 30–40% (by mass) of PM_10_ (Figure 5). With desert dust transport these concentrations tend to decrease while the PM_2.5_/ PM_10_ ratio increases to 35–50%. In regions where DDE contains high anthropogenic pollution, such as DDE from China affecting Japan, this ratio might increase considerably. However, even at receptor sites located thousands of km from emission source, concentrations of PM_10_ and PM_2.5_ are increased to up to several hundreds and tens of μg/m^3^, respectively. Annual mean PM concentrations can reach several hundreds of μg/m^3^ in the emission source region and up to 10 μg/m^3^ at receptor regions. Obviously, all these concentrations vary as a function of the intensity of the episode and distance from the emission source to the receptor region. During transport, dilution increases progressively, thus reducing the contribution of desert dust to local receptor PM. The duration of the episode is also a factor that might affect the dust concentration in the receptor region.

## 5 What is PM from Desert Dust Episodes Made of?

### 5.1 Mineral/Crustal Fraction

The major component of PM during a SDS, around or close to emission sources is the mineral (also known as crustal) fraction (Table S2). Major minerals include quartz, clay (mainly kaolinite, chlorite, and Illite, with lower proportions of montmorillonite-smectite and palygorskite-vermiculite, among others), carbonate (mainly calcite and dolomite, and minor proportions of Fe-bearing carbonates), feldspar and plagioclase, Fe-oxides (goethite, hematite, magnetite), and a variable proportion of salts such as gypsum (Ca-sulfate) and halite (NaCl).^110,129–139^ Freshwater diatom skeletons are also a frequent component of African dust.^140–142^

Journet et al.^135^ and Gonçalves Ageitos et al.^136^ provided estimates of soil mineralogy at a global scale for clay and silt size ranges. Emitted desert dust is enriched in the finest fraction of soil/sediments (clay, < 2 μm, and silt, 2–50 μm, size fractions) and these fractions are enriched in phyllosilicates and iron oxides. In relative concentrations, minerals in PM do not necessarily reflect those of the soil/sediment because the fine fraction of desert dust can be transported during episodic floodings to specific lowlands where emission of desert dust is more frequent and intense.^143^ Journet et al.^135^ found that clay minerals are the main components of the clay and silt fraction of soils/sediments (63–71% in most desert areas and 80–83% in those from North Africa; Figure 6), followed by calcite (2–4% in most areas and 6–9% in Asia, Sahara and Middle East dust; Figure 6). Illite content is believed to be relatively homogeneous, accounting for 25–29% of the clay mineral contents, while those of kaolinite-chlorite are 36–42% in most areas and 49–51% in South African and South American dust. In contrast, smectite makes up 20–26% and 10–11% of clay mineral content in South African and South American dust, respectively. The contents of calcite, and specially quartz and feldspars increase as the size of mineral particles increases. In the desert areas of California, feldspars and clays with relevant contents of quartz and Na-salts, were the prevalent mineral of desert dust emitting sediments, and volcanic glass, with very minor amounts of anorthite in those from Iceland.^139^ Given the high proportions of respirable (PM_4_) crystalline silica (quartz, SiO_2_), acute and persistent exposure to high concentrations of mineral desert dust can at its extreme values, cause silicosis (“desert lung” syndrome).^144^ Text S1 supplies additional information and references on differences of mineralogy among desert dust emitting regions.

**Figure 6:**
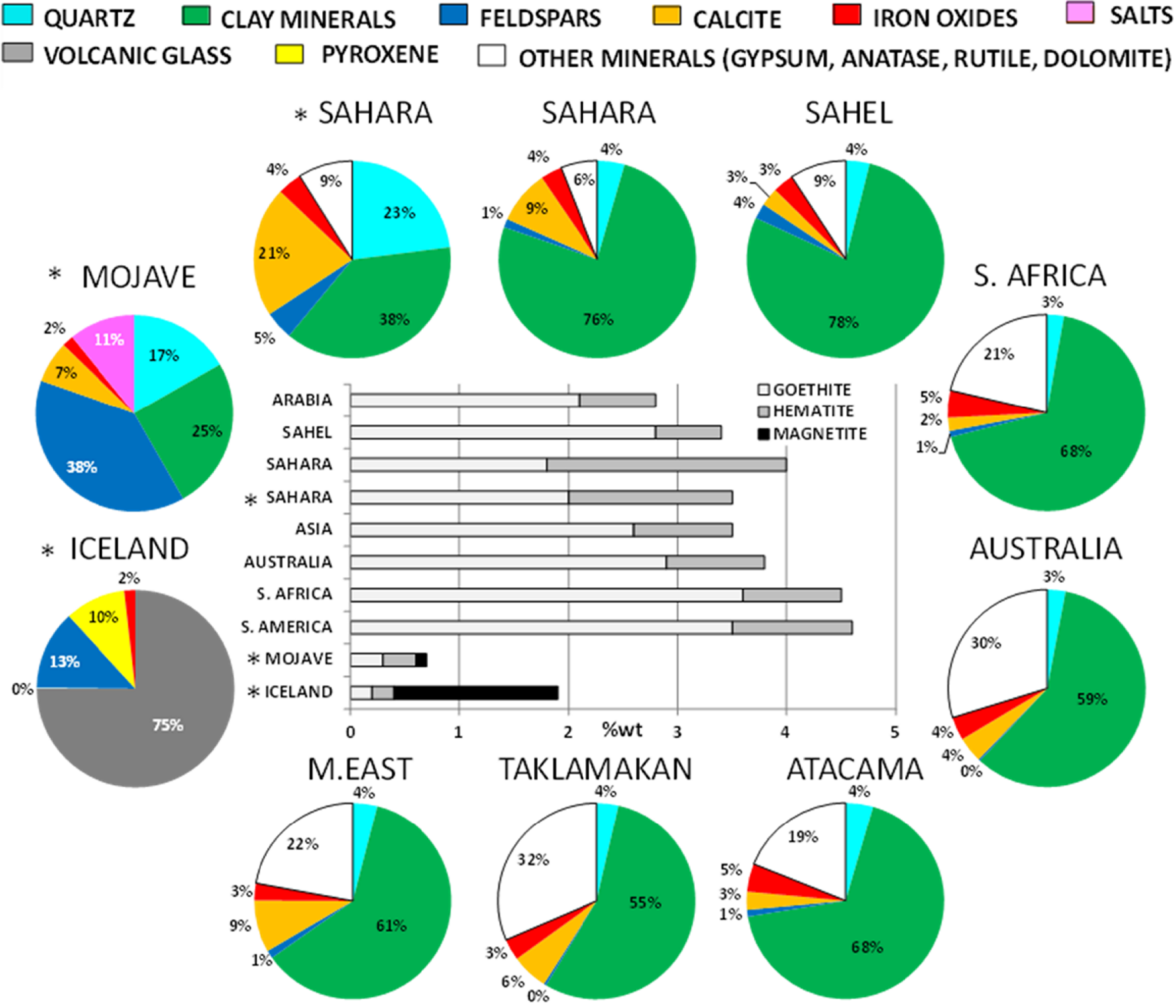
Mineralogy of desert dust from different regions. Central: Content of free iron oxide minerals, in %wt. Surrounding graphs: Content of different mineral groups, in% wt. Modified from Querol et al.^26^ with data from Journet et al.^135^ on mineralogy of desert dust modeled from soil composition; and (*) González-Romero et al.^139^ for soil emitting sediments from Iceland, and California, US, respectively.

### 5.2 Nonmineral Fraction of PM during SDS

During DDE, especially at receptor regions, PM may contain significant proportions of nonmineral desert dust components with proportions increasing with distance from the desert dust emission source. A possible source is sea salt, contributing mainly to Na^+^, Cl^–^, SO_4_^2–^ and Mg^2+^. Anthropogenic pollutants also combine with desert dust in the emission area, during transport, and in the receptor region. Multiple anthropogenic sources situated in desert regions include petrochemical plants, power plants and industrial facilities of North Africa, Northwestern China, and Middle East.^9^ Transport of air masses from highly polluted regions toward or through deserts may interact with desert dust particles. Deposited anthropogenic pollutants can be resuspended with desert dust.^145–149^ Local pollution sources can contaminate aerosolizable material resulting from mineral desert dust.^9,150^ In desert dust receptor regions the PBL height may be markedly reduced during DDEs compared to nondesert dust days resulting in accumulation of local pollution and formation of secondary anthropogenic PM on desert dust particle surfaces.^7,71,151^

These processes may have a major role in changing the properties of desert dust,^152^ including its hygroscopicity^8^ and content of anthropogenic pollutants, such as organic ones, sulfate, nitrate, ammonium, elemental carbon (EC) and locally emitted metals,^110^ which might increase PM health impacts during DDEs. There is a large body of literature on the process of enrichment, reactions, and indirect climate effects, but not on the relevance these might have when evaluating health effects of PM during DDEs.^153,154^

The analysis of long-term PMx (PM_10_, PM_2.5_ or PM_1_) and gaseous pollutants data series revealed very high nondesert dust PM (including locally emitted trace metals CO and NO_2_ concentrations during the most intense DDEs over Barcelona (Northeast Spain).^7,109^ This result was attributed to the thinning of the PBL caused by DDEs. There are also many studies on increased sulfate and nitrate during DDEs.^116,151,155–158^ Similarly, there are multiple studies showing increased organic carbon associated with transported desert dust.^110,159^ Saharan dust might act as a carrier of persistent organic pollutants and metals to the Caribbean.^159,160^ Pesticides may be potentially emitted from desert areas such as the Aral Sea.^161,162^

The organic speciation of desert dust in the free troposphere over the Canary Islands in route to the Caribbean was studied.^163^ This study identified numerous organic species, such as levoglucosan (tracer of biomass burning), dicarboxylic acids (secondary organic aerosols), *n*-alkanes and polycyclic aromatic hydrocarbons (combustion processes), hopanes (road traffic), and secondary organic compounds formed by oxidation of α-pinene and isoprene. The study also detected secondary organic and inorganic species within the desert dust. It has been also found that desert dust can contain organo-nitrogenated and sulfonated compounds.^146^

During DDEs in Mediterranean Europe, within both urban and regional background sites, an enrichment of elemental carbon and elements from local road traffic (Sb, Cu, Zn) and industrial sources (As, Pb, Mn) has been reported, and attributed to the thinning of the boundary layer.^110^ Increased bioreactivity of particles during DDEs in Xi’an, China, due to increased metals from local emissions has also been found.^164^ Inhalable PM during DDEs in Bamako, Mali, was enriched in transition metals and other potentially bioactive and toxic metals/metalloids.^165^ The composition of a DDE plume leaving Western Africa over the Atlantic and showed that the La/Ce/Sm ratios obtained for specific 24h samples^166^ approached ones typical of oil refining using the La-cracking process^167^ rather than typical crustal rare earth elemental ratios. The Fe solubility from desert dust collected over the Canary Islands increased with aging likely due to oxidation of SO_2_ on desert dust particles.^168^ Radionuclides from nuclear accidents (Chernobyl), regions where nuclear tests were previously conducted, and uranium mining areas, also seem to be enriched in desert dust.^169–171^ In Beirut, it was found that the contribution of desert dust to Beirut’s PM composition did not exacerbate its oxidative potential.^149^

The nondesert dust constituents in PMx during DDEs are likely to be more relevant than mineral PM when evaluating health effects across the Western and Central Mediterranean.^172^ Programs monitoring health effects of PM during SDS and DDEs, especially in the latter, should determine the mineral and nonmineral PM concentrations in addition to the bulk PM concentrations to separately and jointly evaluate the health effects for these PM components, and compare effect modification of nondesert dust PM during desert dust days with bulk PM impacts during nondesert dust days.

### 5.3 Bioaerosols in PM during SDS

Goudie^17^ reviewed multiple studies of biological material in desert dust from Kuwait, Iraq, Iran, West Africa, Taiwan, Japan, Korea, Israel, Southern Europe and Turkey between 2006 and 2013. Others were reviewed by Griffin^13^ for prior years. According to multiple studies,^13,17,173,174^ the biomaterial consisted of pollen spores, bacteria, fungi, and viruses that are capable of surviving during long-range transport and thereby globally dispersed.

A recent review concluded that most desert dust events are vehicles of a significant number of pathogenic or opportunistic microorganisms and that originate from large deserts, particularly in Asia and the Sahara.^175^ Microorganisms, such as *Actinobacteria, Bacteroidetes, Firmicutes, Chloroflexi, Cyanobacteria, Gemmatimonadetes, Acidobacteria* (some of which are nonpathogenic), are present in African dust transported toward the Atlantic and Caribbean^125,173,176–178^ and also in Australian dust.^179^ The occurrence of cyanotoxins in the Qatar desert dust was reported.^180^ In Taiwan, ambient air aspergillus fungi peaks were attributed to transported Gobi dust.^181^ Microorganisms and pathogens were identified in winter African dust episodes over the Eastern Mediterranean.^182^ A low microbial biodiversity associated with the African dust was found over Southern Spain, dominated by Firmicutes and Proteobacteria suggesting that transported microbes were alive or present as spores that would germinate under favorable conditions.^183^ These cultivable spores were highly resistant to desiccation, heat, and UV light. Few pathogenic strains were found in the Bodelé dust (Sahel), suggesting that bioaerosol in African dust is not a large threat to public health.^184^ Schuerger et al.^21^ identified science questions and knowledge gaps of microbial transport and survival in Asian and African desert dust plumes reaching North America. A higher bacterial load in PM during dusty days, compared to nondusty ones, was reported in Japan.^185^ In Israel, desert dust storms originating from Middle Eastern dust sources can transport up to 125 times more antibiotic resistance genes than clear atmospheric conditions.^186^

Studies have demonstrated a direct relationship between health effects and bioaerosol load from desert dust storms. In Japan, Watanabe et al.^187,188^ found significant associations between asthma exacerbations and pollen counts in *Kosa* dust air masses that were not apparent during desert dust days without pollen. Data also suggests a relation between desert dust events and infectious disease outbreaks.^175^ Thomson et al.^189^ demonstrated meningococcal contamination in desert dust storms associated with meningitis outbreaks in Africa. *Coccidioides* (*C. immitis* and C. posadasii) fungi in desert dust from the western part of the US cause Valley Fever (coccidioidomycosis or cocci) when inhaled.^190–193^ Anderson^194^ reported 150,000 cases/year in the US; and these numbers are rising due to increased frequencies of desert dust storms and drought conditions. By 2100 in a high warming scenario, it has been predicted that areas of climate-limited endemicity will more than double, the number of affected states will increase from 12 to 17, and the number of Valley Fever cases will increase by 50%.^195^ Dust-Valley Fever outbreaks have been reported in Mexico, Southern US regions, and even Washington State.^196^

The broad spatial pattern and seasonality of meningitis epidemics in the African Sahel^197–199^ and coccidiomycosis epidemics in the US suggest that certain environmental factors, such as low absolute humidity,^199,200^ relative humidity, temperature,^201^ and dusty atmospheric conditions^189,195,197,202–207^ play an important role in the occurrence and spread of these diseases. Identifying the specific climate factor that drives meningitis epidemics is challenging because many environmental variables have prominent seasonal cycles that covary with disease incidence.

## 6 SDS and DDE Monitoring and Alert Systems

As stated by the WHO Air Quality Guidelines,^34^ monitoring and alert systems for dust episodes (both SDS and DDE in this study) should be implemented in desert dust affected regions to (a) alert the population for protective measures; (b) implement measures to reduce PM exposure; and (c) collect air quality data for subsequent health studies.

National desert dust alert and monitoring systems have been implemented in Europe since the late 1990s, when the first EU Daughter Air Quality Directive (1999/30/CE) recognized the relevance of long-range transported desert dust when evaluating PM_10_ concentrations for air quality management. Detailed evaluations of the health effects of PM_1_, PM_1–2.5_ and PM_2.5–10_ and meteorological patterns during DDEs have been made,^172,208^ and alerts sent to (a) populations to take protective measures, and (b) competent administrations to reduce local emissions and abate high PM exposure. Such a tool forecasts and validates the occurrence of desert dust-PM contributions from source to receptor regions. The experience from the EU alert and monitoring systems demonstrates that both accurate modeling tools and surface measurements of anthropogenic and desert dust concentrations of PM_2.5_ and PM_10_ help in assessing health consequences and managing air quality. Below, a structure, built on three main pillars, to develop effective desert dust alert and monitoring systems is proposed.

### 6.1 Modeling to Forecast Desert Dust Episodes and their Transport

Modeling open access products can be used for forecasting/alerting of/on SDSs and DDEs. Examples of these include:

- Regional desert dust alert systems such as the Word Meteorological Organization Sand and Dust Storm Warning Advisory and Assessment Systems for North Africa, Middle East and Europe (https://dust.aemet.es/products/daily-dust-products), Middle East (https://dust.ncm.gov.sa/) or Asia (http://www.asdf-bj.net/).
- Global desert dust alert systems such as the one for desert dust of the Copernicus Atmospheric Monitoring Services (CAMS, https://atmosphere.copernicus.eu/charts/packages/cams/products/aerosol-forecasts)

These services can be used to forecast, alert and subsequently confirm the occurrence of episodes. Information obtained from model products can be incorporated into forecast reports that can be sent 24 h in advance of an event to key air quality stakeholders. These forecasted episodes can then be confirmed with archived modeling data and surface measured PM concentrations and annual lists of SDSs and DDEs can be produced and made publicly available.

### 6.2 Quantitative Analysis of Desert Dust Episodes

Once a list of desert dust-days with impacts on surface PM concentrations in an affected area has been obtained, impacts on surface PM concentrations can be quantified using surface PM measurements and the maximum daily contribution of desert dust to ambient PM_10_ and PM_2.5_ concentrations can be estimated. This requires a network of regional background air quality monitoring (NRBAQM) sites reporting online PM_10_ and PM_2.5_ concentrations to confirm SDSs and DDEs, and calculate net desert dust PM contributions. Net desert dust PM_10_ and PM_2.5_ daily contributions are subtracted from respective bulk PM_10_ and PM_2.5_ concentrations to yield nondesert dust PM_10_ and PM_2.5_ loads (mostly from anthropogenic pollution), following the procedure reported by Pey et al.^99^ If data from NRBAQM sites is not available, net desert dust and nondesert dust loads can be calculated for the individual (evaluated) data sets using the methodology proposed by Barnaba et al.,^209^ but avoiding those from road traffic air quality monitoring site. Currently, the European Commission is updating recommendations produced in 2011 on the procedures to quantify desert dust contributions to PM_10_ and PM_2.5_ over Europe.^210^ The procedure is expected to be published in June 2026.

Other measurements to characterize the bioaerosol load (BPMx), chemical speciation of the PM, PBL depth or thickness of the desert dust layer can be implemented to support the understanding of effects of desert dust on health.

The above protocol allows data sets to be produced with daily net desert dust and nondesert dust (anthropogenic) PMx loads (and BPMx if bioaerosols are monitored) (Figure 7). Epidemiological analyses should consider evaluating health outcomes associated with exposure to dust-PMx, nondesert dust PMx and bulk PMx, and differences of such associations between nondesert dust PMx during desert dust and nondesert dust days.^28^

**Figure 7:**
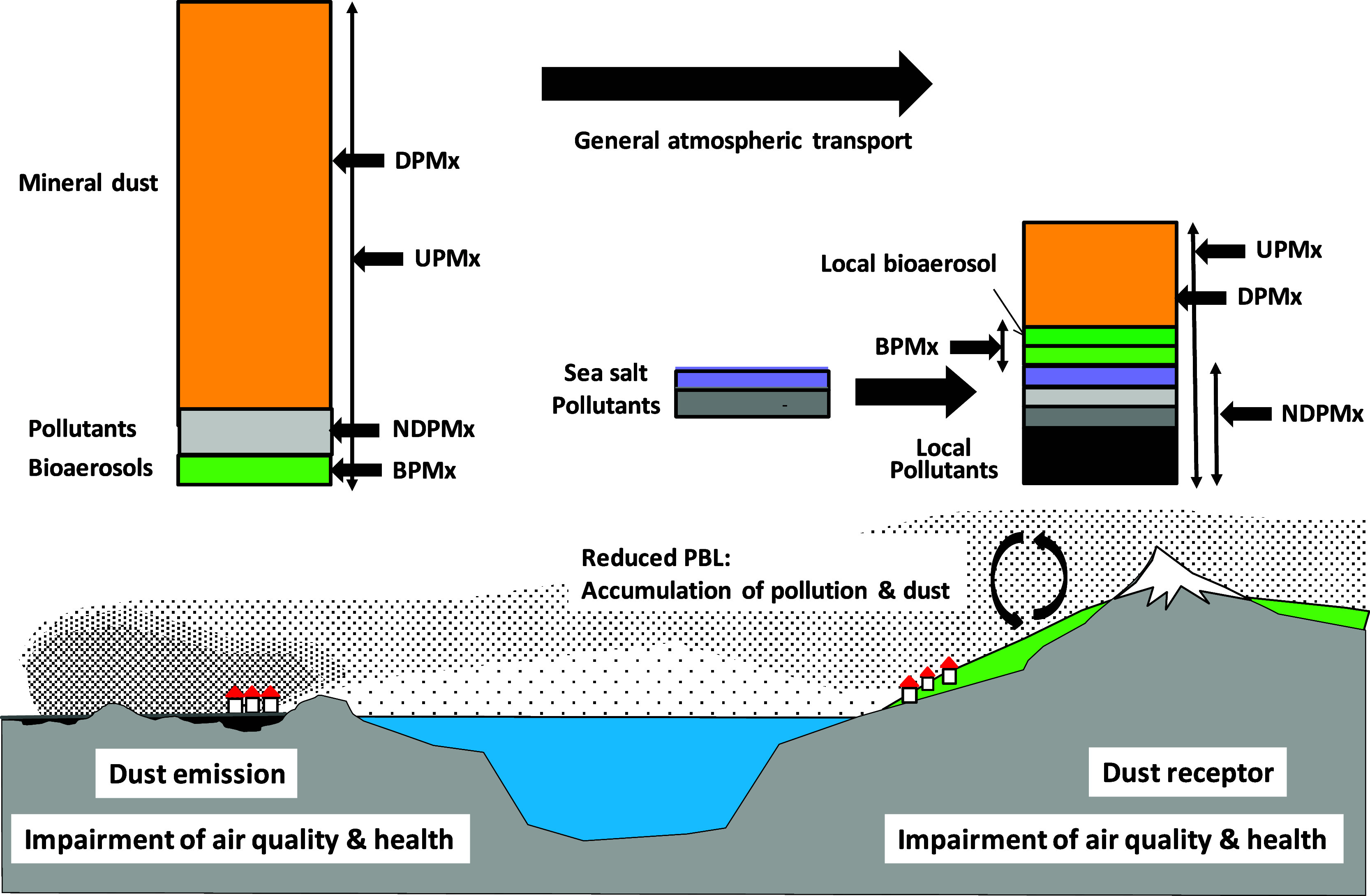
Idealised major PM contributions to be considered during SDS for health studies both at desert dust emitting and desert dust-receptor regions. DPMx represents daily net mineral desert dust PMx; UPMx represents total urban PMx; NDPMx represents nondesert dust PMx (UPMx – DPMx), and BPMx represents biological load in the desert dust. PMx represents PM_10_, PM_2.5_, or PM_1_).

### 6.3 Source Apportionment with Receptor Modeling

Receptor modeling applied to PM speciation data can be very useful in quantifying source contributions to PM. Receptor modeling has been applied to the quantification of the desert dust (and other sources) loads during SDS in emission regions and DDE in the receptor regions by different authors.^164,211–219^

In most studies, this approach is based on sampling and offline chemical analysis of PM, with 1 to 24 h sampling resolution. Recently a number of studies implemented source apportionment of PM using online PM speciation with 1/4 to 1 h resolution.^211,212^ Depending on the source receptor model implemented, a large number of days (usually >100) or hourly chemical PM profiles are required to obtain accurate source apportionment results.

For epidemiological analysis it is necessary to have long data series of source contributions to evaluate short-term effects of exposure to desert dust, and multiannual averages to evaluate the long-term effects. Due to the high costs of these long-term source apportionment studies, multiyear source apportionment data are scarce in both emission and receptor regions.

The most common receptor model used is Positive Matrix Factorization (PMF),^220,221^ of which EPA version 5 ^222^ may be freely downloaded from the US-EPA. When only a few samples are obtained, and the chemical profile of local desert dust and other PM sources are available, the Chemical Mass Balance US-EPA model can also be used for source apportionment.^233^

Most receptor modeling source apportionment studies, Al, Si, Ca, Ti, Fe, and K concentrations are considered as tracers of desert dust.^110,164,223–227^ Furthermore, Cl^–^ is considered a tracer for sea-salt-Na^+^, -Mg^2+^, and – SO_4_^2–^ for sea salt contribution; Vi-Ni and SO_4_^2–^ (and in some cases a low Ce/La rate) for fuel-oil combustion and petrochemical emissions; soluble K^+^ and polysaccharides for biomass burning; As and Se for coal combustion; EC, OC, Cu, Ba, Zn, Sb for road traffic; and Cu, As, Pb, Zn, Fe, Mn for metallurgical emissions; among others.,^110,164^ ^213‑^,^219,224,225,227^

## 7 Recommendations

### 7.1 Major PM and Meteorological Patterns to be Accounted for when Evaluating Health Effects of Desert Dust Exposure

Given the very high concentrations of TSP and PM_10_ attained during intense episodes, absolute concentrations of PM_2.5_ can also increase substantially. It is therefore appropriate to accurately measure concentrations and size distributions when evaluating health effects. Adverse health effects could be the result of mineral desert dust, anthropogenic PM, and/or the biological content associated with desert dust exposure. Recommended approaches to evaluate the association between SDS/DDE and ill health are proposed below.

- Reliable measurements of PM_10_ concentration are essential. Extreme conditions, such as those in the Middle East (high temperature and humidity combined with dust storms), however challenge air quality measurements.^120^ Conventional sampling devices equipped with impaction substrates are not suitable for harsh desert dust storms. Sampler inlets can become clogged and the substrates are prone to particle bounce reducing measurement accuracy.^228^ Dust samplers using a silt acceleration jet and polyurethane as an impaction substrate can remove and retain particles larger than the cut-points (10 and 2.5 um) during desert dust storm events.^229^ High temperature and humidity may limit the ability to conduct fieldwork. For instance, using the gravimetric Harvard Impactors, PM_2.5_ concentrations were found to be 30% lower than the beta attenuation mass and OPSIS optical absorption spectroscopy methods employed by Kuwait EPA.^120^ Desert dust collection efficiency dropped by half as relative humidity increased from 10 to 80%.^230^ Errors resulting during continuous desert dust measurements using glass fiber filters are also evident during high relative humidity.^231^
- If PM_2.5_ is simultaneously measured with PM_10_, the PM_2.5_/PM_10_ ratio provides estimates of fine particulate mass, likely attributable in part to anthropogenic pollution. In some cases, the high mineral PM_10_ load might markedly increase PM_2.5_ concentrations (Figure 8), in which case PM_2.5_/PM_10_ varies as a function of the source area and the transport pathways/duration. Determining the PM_2.5_ contribution enables independent associations of PM_2.5_/PM_2.5–10_ concentrations with health outcomes to be evaluated.
- The health effects of mineral desert dust load in PM_10_ can be directly evaluated by measuring the chemical composition of PM_10_ samples. Online PM_10_ and PM_2.5_ speciation uses modern online X-ray Fluorescence (XRF) analysers that allow concentrations of Si, Al, Fe, Ca, K, Mg, Na, Ti and P to be determined, from which SiO_2_, Al_2_O_3_, Fe_2_O_3_, CaO, MgO, K_2_O, TiO_2_, and P_2_O_5_ can be obtained stoichiometrically, with the sum of these equating to mineral dust concentration.^224,225^ For offline methods, high or low volume PM_10_ and PM_2.5_ samplers can be used for sampling and a subsequent offline analysis. Low volume samplers equipped with Teflon filters and subsequent analysis by XRF is a standard approach because it does not require sample digestion for analysis. Online XRF analyses can supply dust element concentrations on a 15 min to 1 h basis.^211,212^ However, instrument (approximately 150 K€) and maintenance (approximately 10 K€ a year) costs are high. If wet chemistry is used for analysis of PM samples (ICP-AES or ICP-MS), caution is needed in that sample digestion methods for desert dust require use of hydrofluoric acid (HF). Without this silicate and aluminum silicates will dissolve.^210^ When HF is used and evaporated before introducing the sample into the spectrometer, Si is lost. If wet chemistry is the only available tool for chemical analysis, use of quartz microfiber filters, high volume samplers, HF:HNO_3_:HClO_4_ digestion, evaporation of HF, and calculation of SiO_2_ content from the measured Al_2_O_3_*2.5 to 3.0 ^224,225^ is recommended. The use of streaker or DRUM samplers allowing hourly resolution sampling of PM followed by XRF or PIXE analyses also allow offline determination of the concentrations of major desert dust components with 1 h resolution.^226,227,232,233^ Both online and offline procedures can result in large monitoring expenses because of the need to analyze long time-series of daily PM_10_ and PM_2.5_ samples to obtain sufficient data to perform health studies.
- Although the performance of modeling outputs has improved significantly in recent years, uncertainties may still be too large for accurate use in health studies. They do however help to detect SDSs and DDEs (supported by surface PM concentration data) and evaluate source regions of desert dust, transport pathways and thickness and desert dust load of the atmospheric desert dust layer. Models are especially relevant for forecasting SDSs and DDEs to inform the most susceptible population and/or allow preparations for more detailed measurements during a given episode.

**Figure 8:**
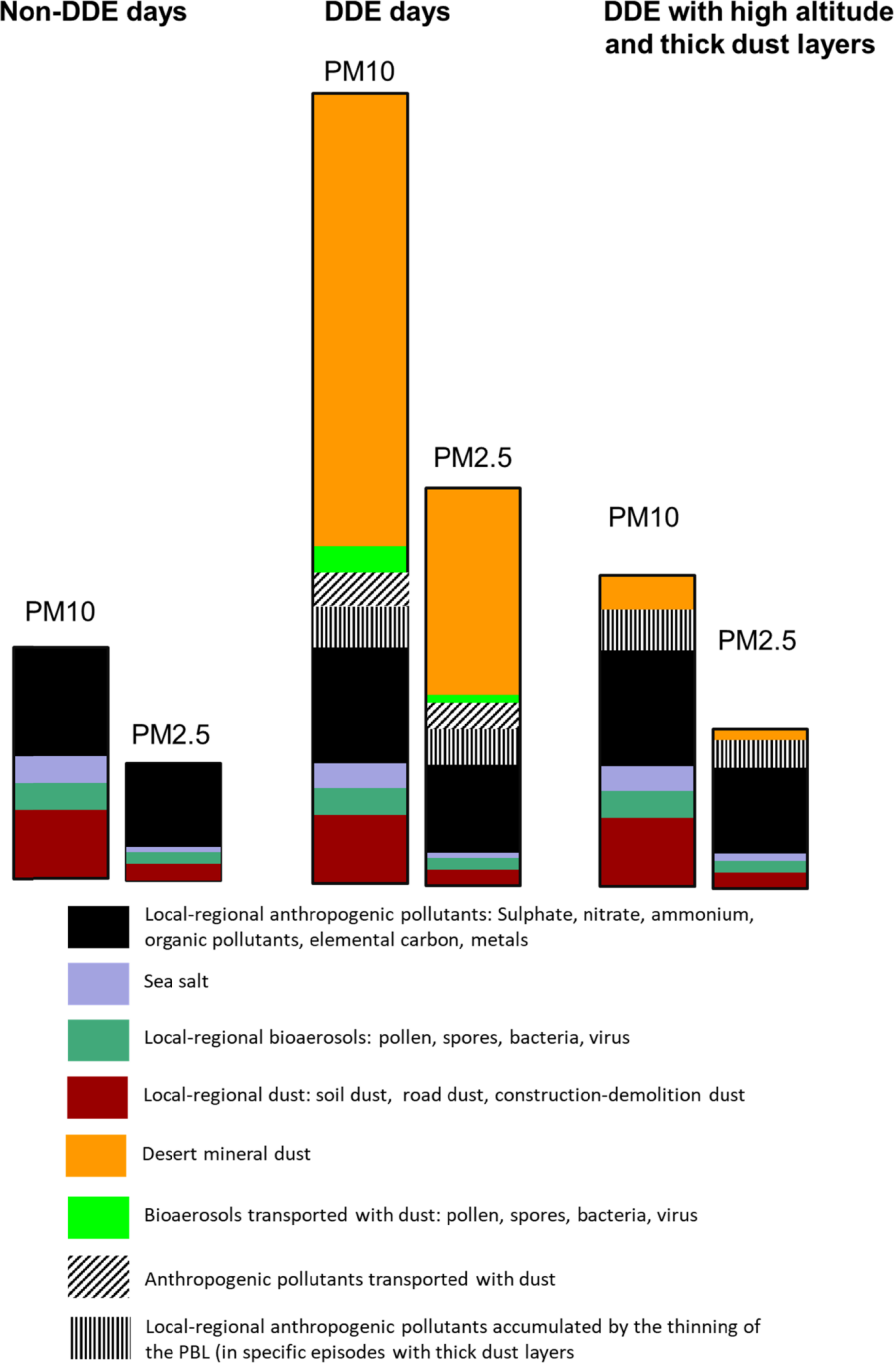
Changes in PM_10_ and PM_2.5_ concentrations and components during days without desert dust episodes, desert dust-days and desert dust-days when the high-altitude thick desert dust layers do not reach the surface levels but decreases PBL and increases local pollution (occurring not necessarily in all DDE). These are idealized PM compositions based on the 2003–2025 continuous measurements performed in receptor sites of Barcelona (urban background) and Montseny (regional background), in NE, Spain. Details can be found in Querol et al.^110^

- Concentrations of inorganic and organic pollutants, with particular relevance to health such as metals and polycyclic aromatic hydrocarbons, should also be analyzed when desert dust arises from highly polluted regions or is transported over such areas before reaching the receptor region (Figure 8).
- Prospero and Lamb^63^ reported a large range of ratios of microorganisms to desert dust concentrations for different DDEs. Therefore, it is also recommended that the biological (fungi, pollen, bacteria, viruses) desert dust load is evaluated for specific areas where it might be relevant for health effects.
- Thick, high altitude atmospheric desert dust air masses over receptor regions stemming from long-range desert dust transport may directly affect surface PM concentrations. They can also indirectly influence surface air quality, by reducing the PBL thickness due to the light scattering of desert dust and/or possible thermal inversions caused by the warm desert dust layer and/or by atmospheric subsidence processes. The subsidence may increase local surface pollutant concentrations irrespective of an effect by the desert dust layer (Figures 8). Models, sounding, or LIDAR measurements can detect the presence of high altitude, thick atmospheric desert dust layers, even if desert dust concentrations at the surface are low. In such cases, it may also be relevant to evaluate the health effects of high anthropogenic PM concentrations.
- Satellite-based data can also be used to estimate ground level PM concentrations.^234,235^ Although satellite coverage is global, polar orbiting satellites only provide snapshots of the aerosol on any given day. With the increasing availability of geostationary satellite data and machine learning tools, it will soon be feasible to obtain data anywhere at any time. While satellite observations provide useful spatial and temporal information regarding large desert dust events, as well as identify affected regions void of monitors, satellites cannot replace ground-based monitoring to adequately characterize desert dust impacts on surface air quality, and satellites may miss desert dust events depending on the timing of satellite coverage.^236^

### 7.2 Challenges and Limitations of Monitoring PM in Desert Areas

Monitoring and reporting PM concentrations in the Gulf Cooperation Council countries is complicated by various challenges, including high temperatures, humidity and background concentrations of fine desert dust most of the year, and occasional very high desert dust loading during SDS events. The latter causes instruments to reach their maximum measurement limits rapidly, after which it becomes difficult to assess the accuracy of desert dust measurements. For example, consecutive major desert dust storms occurred across the Middle East resulting in aerosol optical depths greater than 2.5, reducing surface solar radiative energy, ∼ 350 W m^–2^ drop in the downward shortwave radiation flux in Abu Dhabi, and likely substantial health impacts.^237^

- To mitigate these factors and ensure valid data capture and analysis, stringent procedures must be followed prior to an SDS to ensure instruments operate optimally. For beta gauge instruments, to avoid flow variation or heavy desert dust loading that could result in clogging and/or tearing of the filter, the instrument is reprogrammed to reduce the measurement cycle timing from 24 h per spot to 3 h and the measurement period timing (reading) maintained every 1 h. This ensures that relatively minimal desert dust is accumulated on each spot, and that the instrument automatically does an initial check every hour to assess any clogging or problems in the pumping system. This process prevents/minimizes instrument malfunction or failure thus minimizing uncertainty as to whether high concentration readings are due to desert dust from SDS or from clogging and instrument malfunction. Although it is a constant challenge, through years of experience, operators in the region can anticipate what issues may arise during SDS and act accordingly to minimize data loss. After the storm, a stringent cleaning and maintenance of the equipment is crucial to return the stations to their optimal operation.
- High temperatures and humidity affect all monitoring technologies. Abu Dhabi regularly experiences summer temperatures above 40 °C, therefore stations need to be cooled by air conditioning to protect instruments from overheating and malfunctioning. The upper operating temperature limit for the PM instrument is 40 °C.^238^ The difference in temperatures outside and inside the stations causes condensation in sampling lines which has been observed to result in higher readings.

## 8 Conclusions

To properly assess the health effects of SDS in and around desert dust emitting areas and DDE in receptor regions, accurate exposure data are essential.^33^ Accordingly, the need to implement scientifically sound and harmonized protocols to evaluate health effects of SDS and DDE is highlighted repeatedly.^29,33,34^ These protocols must take account of contributions from high concentrations of desert dust, associated bioaerosols (bacteria, spores, viruses) and anthropogenic pollutants.

Not only are PM concentrations much higher in SDSs compared to DDEs, but the relative load of desert dust and nondesert dust in PM might be different. In many studies conducted in receptor regions, the nondesert dust PM fraction on desert dust days has been linked with negative health outcomes that are more harmful than PM of nondesert dust days. Also, in receptor regions, thick (up to 7 km) atmospheric layers of desert dust cause decreased solar radiation on the surface such that convective atmospheric dynamics during daylight are reduced compared to nondesert dust days, and the mixing layer is markedly reduced in thickness. Under this scenario, local pollutants become highly concentrated and mixed with desert dust.

Thus, mineral desert dust is not the only component of PM that may affect health during SDSs and DDEs, but other patterns of PM and atmospheric conditions need to be considered. Tools have been successfully implemented to monitor these patterns, parameters, contributions, and loads. Forecast and alert systems based on modeling tools can provide relevant semiquantitative data on surface concentrations for mineral desert dust in receptors and emitting regions to protect populations and identify desert dust days. Complemented with harmonized surface measurements that collect data that includes mineral desert dust and nondesert dust PM concentrations, their compositions, combined with data sets on key health relevant parameters may supply adequate information for implementing epidemiological studies on health effects of SDSs and DDEs.

Quantitative outputs of modeling tools on desert dust surface concentration have uncertainties that avoid the direct use in scientifically sound statistically based epidemiological studies. However, this may change, with services such as CAMS, which are improving outputs and promise to provide reanalysis products for desert dust and other PM compounds in the near future. These products would significantly improve current forecast. The provision of such reanalysis products is currently under progress. SDSs and DDEs are complex processes that require a specific multidisciplinary approach to evaluate their health effects.

The complexity resides both in understanding and characterizing different PM components and meteorological patterns when evaluating environmental exposures and implementing adequate epidemiological hypotheses and designs to evaluate health effects. Efforts should focus on a transdisciplinary approach, bridging the atmospheric and epidemiological communities, and their collaboration with toxicologists, meteorologists, air quality measurement communities and modelers. Studies are now addressing the application of machine learning and artificial intelligence to SDS and DDE.^239–244^ The capabilities of these tools are encouraging for use in the near future to improve methodologies that detect, predict and describe SDS and DDE, quantify desert dust contributions and in doing so, support the evaluation associated health effects.

## Supplementary Material



## Notes

Figure showing the main surface impact of dust in PM concentrations of the continental areas; a table with usual PM concentration ranges during dust episodes near source regions; a table summarizing mineralogical composition of dust from different regions, and text describing major differences (PDF)
