# Supplementary material for: Desert Sand and Dust Storms and Desert Dust Episodes: Major Patterns to be Accounted for to Protect the Health of Exposed Population: A Review
Source: ACS EST Air. 2025 Nov 24;2(12):2731–52. doi: 10.1021/acsestair.5c00201 (PMC12706757; doi:10.1021/acsestair.5c00201)
Supplement: Supplementary file 1 [file ea5c00201_si_001.pdf]

Desert sand and dust storms, and desert dust episodes: major patterns to be accounted for to protect the health of exposed population: A review

Xavier Querol[1], Julia Fussell[2], Najat A. Saliba[3], Ali Al-Hemoud[4], Kari C. Nadeau[5], Aurelio Tobías[1], Masahiro Hashizume[6], Mazen Malkawi[7] Sophie P. Gumy,[8] Kerolyn K. Shairsingh[8] Pierpaolo Mudu[9] Philip K. Hopke[10,11]

1. Institute of Environmental Assessment and Water Research (IDAEA), Spanish National Research Council (CSIC), Barcelona, 08034, Spain
2. Environmental Research Group, Medical Research Council (MRC) Centre for Environment and Health, School of Public Health, Imperial College London, London, SW7 2AZ, United Kingdom
3. Member of the Lebanese Parliament, Beirut, VGW3+QJ3, Lebanon
4. Kuwait Institute for Scientific Research, Environment and Life Sciences Research Centre, Safat, 13109, Kuwait
5. Department of Environmental Health, Harvard T.H Chan School of Public Health, Boston, 02115, MA, USA
6. Department of Global Health Policy, Graduate School of Medicine, The University of Tokyo, 113-0033, Japan
7. Environmental Health Advisor at the Ministry of Health and Prevention, P.O.1853, Dubai UAE
8. Department of Environment, Climate Change and Health, World Health Organization, Geneva, 1211, Switzerland
9. World Health Organization, Regional Office for Europe, European Centre for Environment and Health, Bonn, D-53113, Germany
10. Institute for a Sustainable Environment, Clarkson University, Potsdam, NY, 13699, USA
11. Departments of Public Health Sciences and Environmental Medicine, University of Rochester School of Medicine and Dentistry, Rochester, 14642, NY, USA

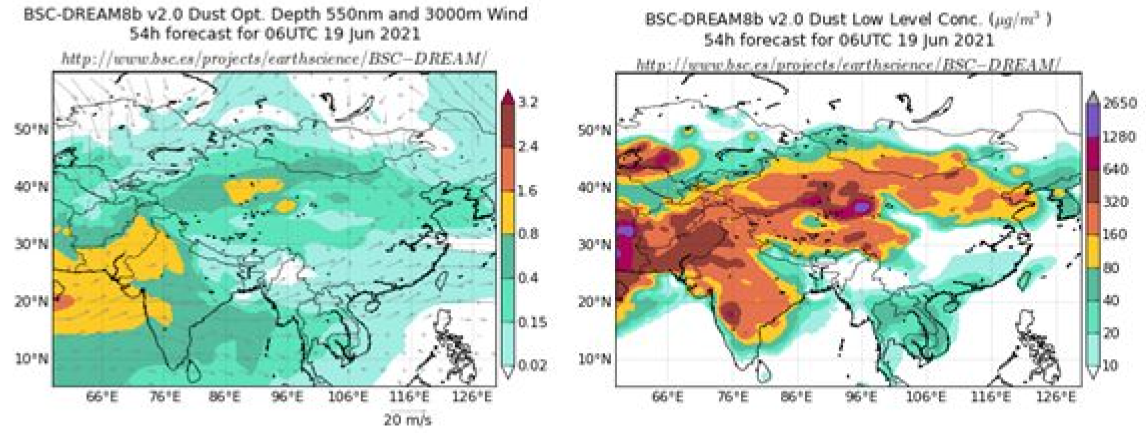

Figure S1. BSC-DREAM8B modelling outputs for dust episodes over Asia on 19th June 2021. The aerosol optical depth (left panel) covers large extensions of the ocean around India. The surface dust impact (right panel) only extends on the continent and a short way along the coasts. The thinner marine PBL caused isolation of the dust from upper layers from surface levels. However, in the continent, the growing of the PBL caused dust transport to surface levels (right).

Table S1. Near source region PM concentrations from selected studies

Location	Period	Species	Peak Concentrations (μg/m^3)	Reference
Inner Mongolia	March 2001 (8 h)	TSP	6700	Mori et al., 2003
Beijing	March 2001 (6 h)	TSP (93% between 2.1-20 μm)	1500	Mori et al., 2003
Remote Island, Japan	March 2001 (24 h)	TSP (64% between 2.1-20 μm)	230	Mori et al., 2003
Tehran, Shiraz, Isfahan, Arak, Ahvaz, and Tabriz, Iran	2009–2010	PM_{10}	104 to 2918	Sotoudeheian et al. (2016)
Beer-Sheva, Negev, Israel	2001-2012	PM_{10}	122[a]	Krasnov et al., 2014
Sydney Australia	September 2009	PM_{10}	11,800	Aryal et al., 2012
Beirut, Lebanon	August 2009 and April 2010	PM_{10}	105[b]	Saliba and Chamseddine, 2012
Beirut, Lebanon	August 2009 and April 2010	$PM_{2.5}$	34[b]	Saliba and Chamseddine, 2012
Kuwait City, Kuwait	2017-2019	PM_{10}	152[b]	Alahmad et al., 2021
Kuwait City, Kuwait	2017-2019	$PM_{2.5}$	54[b]	Alahmad et al., 2021
Tinfou, Morocco	2006	TSP	300,000	Kandler et al., 2009
Tinfou, Morocco	2006	PM_{10}	3,000	Kandler et al., 2009
Tinfou, Morocco	2006	$PM_{2.5}$	1,000	Kandler et al., 2009
Cape Verde	January and February 2008	TSP	542	Kandler et al., 2011
Cape Verde	January and February 2008	PM_{10}	460	Kandler et al., 2011
Cape Verde	January and February 2008	$PM_{2.5}$	184	Kandler et al., 2011
Tenerife, Canary Islands, Spain	February-March 2000	PM_{10}	675	Viana et al..2002
Hotan, China	2000–2013	PM_{10}	352[c]	Zhang et al., 2017

a. Daily net contribution of dust calculated for the entire period.

b. Mean value during 19 high dust days.

c. Annual average over the whole period.

*Table S2. Minerals typically present in desert dust according to the references reported in the text. Asterisks indicate abundance: ***** very high (tens of %wt) to * low (less than 1%wt). Modified from Querol et al. (2019a) and González-Romero et al. (2024).*

Silicate & aluminum-silicate minerals	Silicates	Quartz	SiO_2 (mineral grains or diatomea fragments)	*****
	Clay minerals	Kaolinite	$Al_2Si_2O_5(OH)_4$	****
		Illite	$(K,H_3O)(Al,Mg,Fe)_2(Si,Al)_4O_{10}[(OH)_2,(H_2O)]$	****
		Chlorite	$((MgFe)_5Al)(AlSi_3)O_{10}(OH)_8$	***
		Palygorskite	$(Mg,Al)_2Si_4O_{10}(OH)\cdot 4(H_2O)$	***
		Montmorillonite	$(Na,Ca)_{0.33}(Al,Mg)_2(Si_4O_{10})(OH)_2\cdot nH_2O$	***
	Feldspars	Albite	$NaAlSi_3O_8$	**
		Anorthite	$CaAl_2Si_2O_8$	**
		Microcline/orthoclinic.	$KAlSi_3O_8$	**
	Other silicates	Zircon	$ZrSiO_4$	*
		Hornblende	$Ca_2(Mg,Fe,Al)_5(Al,Si)_8O_{22}(OH)_2$	*
Carbonate minerals	Ca & Mg carbonate	Calcite	$CaCO_3$	****
		Dolomite	$(CaMg)_2CO_3$	**
Oxide minerals	Iron oxides	Hematite	Fe_2O_3	**
		Magnetite	Fe_3O_4	*
		Goethite	α-FeO(OH)	**
	Other oxides	Anatase & rutile	TiO_2	*
Salts	Chlorides	Halite	NaCl	*
	Sulfates	Gypsum	$CaSO_4.2H_2O$	**
		Thenardite	Na_2SO_4	*
		Epsomite	$MgSO_4$	*
Phosphate minerals		Apatite	$Ca_5(PO_4)_3(F,Cl,OH)$	*
**Dust from Iceland**				
	Volcanic Glass	Amorphous glass	Si-Al-Ca-Fe-Mg-O	*****
	Feldspars	Anorthite	$CaAl_2Si_2O_8$	***
	Iron oxides	Magnetite	Fe_3O_4	**
	Silicates	Quartz	SiO_2	*

Text S1. Regional differences in dust composition

In addition to these general patterns presented in Section 5, there are important regional differences in mineral dust composition. A number of studies on mineralogy of desert dust and soil/sediments (e.g., Marsden et al.[1]), found that kaolinite, quartz and hematite prevail in the dust composition of the Sahel region, whereas illite-palygorskite, quartz, and calcite dominate in the Saharan dust. Shen et al.[2] found that illite, kaolinite (around 47-52% total clay content), quartz (25-27%), feldspar and plagioclase (6-7%), calcite and dolomite (13-18%), with traces of gypsum, hornblende (an Al-silicate), and halite were the main components of NE China desert dust. Engelbrecht et al.[3,4] reported that dust from Middle East-Central Asia was also made up of quartz, other silicate minerals, carbonates, oxides, sulphates, and salts in various proportions. In comparison with the Sahara, China, US, and world dusts[5,6], Middle East samples had lower SiO_2, Fe- and Mn-oxides and P_2O_5 contents and higher CaO and MgO contents (the latter being present as carbonate minerals). Among seven global dust zones, dust in the northern Arabia (Jordan, Iraq, Kuwait, Saudi Arabia, Bahrain, Qatar) was reported to have the highest amounts of carbonates (43%) and the second lowest amounts of quartz (37%) and feldspar (14%)[7]. Aryal[8] reported quartz, anatase (TiO_2), calcite, feldspars, halite, hematite, and clays (kaolinite, illite-muscovite and montmorillonite) as major components of Australian desert dust. For desert dust (PM20 in this case) of Arizona, US, Ghio et al.[9] reported a prevalence of clay (47-63%, illite, smectite and kaolinite), quartz (15-19%), carbonate minerals (13-23%, calcite and dolomite), feldspars (6-9%, microcline and plagioclase) and iron oxides (0-7% hematite and goethite).

Furthermore, there are specific regions where mineral dust composition is totally different to that reported above for a large number of desert areas. In Iceland, source rocks are made up of relatively recent volcanic materials and dust derived from the erosion of these have a completely different mineralogy to those having old sedimentary and metamorphic geological substrata. Baldo et al.[10] found that Iceland desert dust consists of 8-92 (most 60-92)% amorphous glassy-volcanic materials. Anorthite (Ca-plagioclase, 4-46%) and a pyroxene (augite, 4-30%) are the major crystalline mineral phases, with minor olivine (forsterite, 0-7%), and K-feldspar (microcline, 0-10%) the minor ones and in addition, trace amounts of (titano)magnetite are found. Furthermore, compared to other desert dust a high Fe content (10–13%), with a relevant fraction of Fe oxides occurring as magnetite (1-2% of dust), but most (80-90%) occurring in glass and pyroxenes.

1. Marsden, N. A., Ullrich, R., Möhler, O., Eriksen Hammer, S., Kandler, K., Cui, Z., Williams, P. I., Flynn, M. J., Liu, D., Allan, J. D., and Coe, H., 2019. Mineralogy and mixing state of north African mineral dust by online single-particle mass spectrometry, Atmos. Chem. Phys., 19, 2259–2281, https://doi.org/10.5194/acp-19-2259-2019.
2. Shen Z. Caquineau S., Cao J., Zhang X., Hana Y., Gaudichet A., Gomes L., 2009. Mineralogical characteristics of soil dust from source regions in northern China. Particuology 7, 507-512.

3. Engelbrecht J.P., McDonald E.V., Gillies J.A., Gertler A.W., 2008. Department of Defense Enhanced Particulate Matter Surveillance Program (EPMSP). Final Report. Desert Research Institute Reno, NV, US, 57 pp.
4. Engelbrecht J.P., McDonald E.V., Gillies J.A., Jayanty R.K.M., Casuccio G., Gertler A.W., 2009. Characterizing Mineral Dusts and Other Aerosols from the Middle East—Part 1: Ambient Sampling. Inhalation Toxicology 21 , 4, 297-326.
5. Goudie A.S., Middleton N.J., 2006. Desert dust in the global system. Springer, Heidelberg. ISBN 978-3-540-32355-6, 288 pp.
6. Labban R., Veranth. M., Chow J.C., Engelbrecht J., Watson J.G., 2004. Size and geographical variation in PM1, PM2.5, and PM10: Source profiles from soils in the western United States. Water Air Soil Poll. 157, 13-31.
7. Al-Dousari, A., Al-Awadhi, J. Ahmed, M., 2013. Dust fallout characteristics within global dust storm major trajectories. Arabian Journal of Geosciences. 6, 3877-3884.
8. Aryal R., Kandel D., Acharya D., Chong M.N., Beecham S., 2012. Unusual Sydney dust storm and its mineralogical and organic characteristics. Environ. Chem. 9, 537-546.
9. Ghio, A.J., Kummarapurugu, S.T., Tong, H., Soukup, J.M., Dailey, L.A., Boykin, E., et al., 2014. Biological effects of desert dust in respiratory epithelial cells and a murine model. Inhal. Toxicol. 26, 299–309.
10. Baldo, C., Formenti, P., Nowak, S., Chevaillier, S., Cazaunau, M., Pangui, E., Di Biagio, C., Doussin, J.-F., Ignatyev, K., Dagsson-Waldhauserova, P., Arnalds, O., MacKenzie, A. R., Shi, Z., 2020. Distinct chemical and mineralogical composition of Icelandic dust compared to northern African and Asian dust, Atmospheric Chemistry and Physics, 20, 21, 13521—13539.
